# Dipeptidyl Peptidase-4 (DPP-4) Inhibitors Are Associated With Reduced Early but Increased Chronic Postoperative Opioid Use After Shoulder Surgery

**DOI:** 10.7759/cureus.110873

**Published:** 2026-06-15

**Authors:** Ronak J Mahatme, Shawn A Moore, Anish Gangavaram, Nishanth Muthusamy, Esha Reddy, David L Bernholt, Brian M Grawe

**Affiliations:** 1 Department of Orthopedics, University of Cincinnati College of Medicine, Cincinnati, USA; 2 Department of Orthopedic Surgery, University of Cincinnati College of Medicine, Cincinnati, USA

**Keywords:** dipeptidyl peptidase 4 (dpp-4), ed visits, postoperative opioid use, shoulder surgery, type ii diabetes

## Abstract

Background

Diabetes mellitus increases the risk of postoperative complications following orthopedic shoulder surgery. Dipeptidyl peptidase-4 (DPP-4) inhibitors are widely prescribed for glycemic control in type 2 diabetes mellitus, yet their influence on perioperative and long-term postoperative outcomes remains unclear.

Methods

A retrospective cohort study was conducted using the TriNetX Research Network to identify adults (≥18 years) with type 2 diabetes who underwent orthopedic shoulder procedures and were either prescribed or not prescribed a DPP-4 inhibitor. After 1:1 propensity score matching for demographics, comorbidities, and procedure type, 834 patients were included in each cohort. Outcomes included 30-day emergency department (ED) visits, one- and two-year reoperation rates, and postoperative opioid use.

Results

ED visits and reoperation rates at one and two years were similar between cohorts. DPP-4 inhibitor users demonstrated significantly lower rates of early postoperative opioid use compared with non-users (33.6% vs. 45.3%; RR: 0.741; 95% CI: 0.656-0.836; p < 0.001). No significant difference was observed in prolonged opioid use (21.5% vs. 21.2%; RR: 1.011; 95% CI: 0.841-1.216; p = 0.952). However, DPP-4 users had significantly higher rates of chronic postoperative opioid use (21.2% vs. 13.7%; RR: 1.553; 95% CI: 1.252-1.925; p < 0.001).

Conclusion

DPP-4 inhibitor exposure within six months prior to shoulder surgery was not associated with differences in long-term surgical outcomes or early emergency medical utilization among patients with type 2 diabetes. However, DPP-4 users demonstrated lower early but higher chronic postoperative opioid use, suggesting that incretin pathway modulation may have time-dependent effects on postoperative pain physiology. These findings suggest a potential link between metabolic therapy and postoperative pain adaptation and warrant further investigation into the underlying mechanisms.

## Introduction

Type 2 diabetes mellitus (T2DM) affects approximately 6% of the global population and continues to rise in prevalence worldwide [1,2]. Beyond its metabolic effects, diabetes has been consistently associated with higher rates of postoperative complications across surgical specialties [3,4]. Impaired glucose metabolism contributes to chronic inflammation, endothelial dysfunction, and delayed tissue repair, all of which increase risks of infection, wound complications, and prolonged hospitalization following orthopedic procedures [5,6]. 

In shoulder surgery, diabetic patients exhibit elevated rates of complications such as adhesive capsulitis, rotator cuff retear, and periprosthetic infection [7-11]. These issues not only hinder recovery but also increase reoperation rates and postoperative pain medication use. Evidence suggests that tighter perioperative glycemic control mitigates these risks, underscoring the importance of optimizing antidiabetic therapy before surgery [12,13]. 

Dipeptidyl peptidase-4 (DPP-4) inhibitors-including sitagliptin, saxagliptin, and linagliptin-are oral antihyperglycemic agents that enhance incretin activity to stimulate insulin secretion and suppress glucagon release [14,15]. Beyond glycemic regulation, DPP-4 inhibitors exhibit anti-inflammatory and vasculoprotective effects, which may influence surgical recovery. Despite their widespread use, the perioperative impact of DPP-4 therapy on orthopedic outcomes remains poorly defined. 

The objective of this study was to evaluate postoperative outcomes in patients with T2DM undergoing shoulder surgery who were treated with DPP-4 inhibitors compared with diabetic patients not receiving DPP-4 therapy, using a large national health research database. We hypothesized that DPP-4 inhibitor use would be associated with comparable or improved postoperative outcomes, reflecting potential metabolic and anti-inflammatory benefits of incretin pathway modulation. 

## Materials and methods

The data used in this retrospective cohort study were collected on October 1st, 2025, from the TriNetX Research Network [16], which provided access to electronic medical records (diagnoses, procedures, medications, laboratory values, genomic information) from over 150 million patients from 102 healthcare organizations. TriNetX, LLC (Cambridge, MA, USA) [16] is compliant with HIPAA and applicable data privacy regulations, and all data accessed through the platform is de-identified in accordance with federal standards. Because this study used only de-identified patient records and did not involve the collection, use, or transmittal of individually identifiable data, this study was exempted from Institutional Review Board approval.

Adult patients (≥18 years) with T2DM who underwent non-diagnostic shoulder arthroscopy or open orthopedic procedures for repair, reconstruction, fracture fixation, or dislocation treatment between October 1, 2003, and October 1, 2023, were included. The DPP-4 cohort comprised patients with documented DPP-4 inhibitor exposure within six months prior to surgery. Postoperative medication records were assessed to characterize exposure patterns but were not used to define cohort assignment. The non-DPP-4 cohort included patients without documented DPP-4 inhibitor exposure during the study period. Exclusion criteria included polytrauma within six months of surgery, type 1 diabetes mellitus, systemic connective tissue disorders, long-term corticosteroid use, autoinflammatory syndromes, inflammatory polyarthropathies, prior stress or pathological shoulder/humerus fractures, and previous shoulder surgery.

Propensity score matching was performed 1:1 for age, sex, body mass index, tobacco use, comorbidities, osteoporosis, procedure type, metformin use, and insulin use. After matching, both cohorts included 834 patients (Table 1). Prior to matching, the DPP-4 cohort differed substantially from the non-DPP-4 cohort across several clinically meaningful characteristics. DPP-4 inhibitor users had significantly higher rates of hypertensive disease (86.9% vs. 77.1%), dyslipidemia (80.5% vs. 67.1%), diabetic kidney complications (19.3% vs. 7.2%), and diabetic neurological complications (16.5% vs. 8.4%), as well as higher rates of concurrent metformin (84.9% vs. 39.8%) and insulin use (61.6% vs. 31.8%). These differences reflect the known prescribing pattern of DPP-4 inhibitors, which are typically reserved for patients with more advanced or refractory T2DM who require combination antihyperglycemic therapy and have higher rates of end-organ involvement. The non-DPP-4 cohort, by contrast, represented a broader diabetic population with comparatively lower comorbidity burden. Propensity score matching effectively balanced these differences, yielding well-matched cohorts across all covariates (Table 1).

**Table 1:** Propensity score matching for patients with type 2 diabetes mellitus undergoing orthopedic shoulder procedures DPP-4 - dipeptidyl peptidase-4; BMI - body mass index; CKD - chronic kidney disease

Characteristic	Before matching				After matching			
	DPP-4 inhibitor (% of cohort)	Non-DPP-4 inhibitor (% of cohort)	p-value	Std. Diff.	DPP-4 inhibitor (% of cohort)	Non-DPP-4 inhibitor (% of cohort)	p-value	Std. Diff.
Total	835 (100%)	34,932 (100%)			834 (100%)	834 (100%)		
Demographics								
Age at index	63.2 ± 10.0 (100%)	60.9 ± 10.7 (100%)	<0.001	0.223	63.2 ± 10.0 (100%)	62.9 ± 10.1 (100%)	0.497	0.033
Female	382 (45.7%)	16,035 (46.1%)	0.846	0.007	381 (45.7%)	387 (46.4%)	0.768	0.014
Male	453 (54.3%)	18,745 (53.9%)	0.83	0.008	453 (54.3%)	446 (53.5%)	0.731	0.017
Diagnosis								
Osteoporosis without current pathological fracture (M81)	46 (5.5%)	1870 (5.4%)	0.865	0.006	46 (5.5%)	42 (5.0%)	0.661	0.021
BMI 30–39, adult (Z68.3)	142 (17.0%)	6657 (19.1%)	0.122	0.055	142 (17.0%)	142 (17.0%)	0.308	0.05
BMI 40+, adult (Z68.4)	54 (6.5%)	3246 (9.3%)	0.005	0.106	54 (6.5%)	53 (6.4%)	0.92	0.005
Tobacco use (Z72.0)	30 (3.6%)	1537 (4.4%)	0.251	0.042	30 (3.6%)	21 (2.5%)	0.201	0.063
Heart failure (I50)	57 (6.8%)	2433 (7.0%)	0.852	0.007	57 (6.8%)	55 (6.6%)	0.845	0.01
Hypertensive diseases (I10–I1A)	726 (86.9%)	26,823 (77.1%)	<0.001	0.259	725 (86.9%)	725 (86.9%)	0.884	0.007
Disorders of lipoprotein metabolism and other lipidemias (E78)	672 (80.5%)	23,357 (67.1%)	<0.001	0.307	671 (80.5%)	683 (81.9%)	0.452	0.037
Chronic ischemic heart disease (I25)	181 (21.7%)	6799 (19.5%)	0.125	0.053	181 (21.7%)	201 (24.1%)	0.244	0.057
Type 2 diabetes mellitus with kidney complications (E11.2)	161 (19.3%)	2515 (7.2%)	<0.001	0.361	161 (19.3%)	150 (18.0%)	0.489	0.034
Type 2 diabetes mellitus with neurological complications (E11.4)	138 (16.5%)	2904 (8.4%)	<0.001	0.247	137 (16.4%)	121 (14.5%)	0.279	0.053
Procedure								
Muscle transfer, any type, shoulder or upper arm (1004202)	10 (1.2%)	187 (0.5%)	0.011	0.071	10 (1.2%)	10 (1.2%)	1	<0.001
Tenotomy, shoulder area (1004206)	12 (1.4%)	344 (1.0%)	0.198	0.04	12 (1.4%)	10 (1.2%)	0.668	0.021
Arthroscopy, shoulder, surgical (1005614)	528 (63.2%)	24,267 (69.7%)	<0.001	0.138	528 (63.3%)	549 (65.8%)	0.282	0.053
Repair of ruptured musculotendinous cuff, open (1004209)	67 (8.0%)	2742 (7.9%)	0.88	0.005	67 (8.0%)	51 (6.1%)	0.127	0.075
Capsulorrhaphy, anterior (1004216)	0 (0%)	52 (0.1%)	0.264	0.055	0 (0%)	0 (0%)	--	--
Capsulorrhaphy, anterior, any type (1004219)	0 (0%)	35 (0.1%)	0.359	0.045	0 (0%)	0 (0%)	--	--
Arthroplasty, glenohumeral joint (1004224)	217 (26.0%)	5638 (16.2%)	<0.001	0.242	216 (25.9%)	200 (24.0%)	0.365	0.044
Prophylactic treatment — nailing, pinning, plating or wiring w/ or w/o methylmethacrylate (1004229)	10 (1.2%)	50 (0.1%)	<0.001	0.129	10 (1.2%)	0 (0%)	0.002	0.156
Osteotomy, clavicle, w/ or w/o internal fixation (1014575)	0 (0%)	93 (0.3%)	0.135	0.073	0 (0%)	0 (0%)	--	--
Scapulopexy (1023400)	0 (0%)	10 (0.0%)	0.624	0.024	0 (0%)	0 (0%)	--	--
Coracoacromial ligament release, w/ or w/o acromioplasty (23415)	10 (1.2%)	143 (0.4%)	0.001	0.088	10 (1.2%)	10 (1.2%)	1	<0.001
Reconstruction of complete shoulder (rotator) cuff avulsion, chronic, including acromioplasty (23420)	10 (1.2%)	626 (1.8%)	0.194	0.05	10 (1.2%)	10 (1.2%)	1	<0.001
Tenodesis of long tendon of biceps (23430)	150 (18.0%)	4650 (13.4%)	<0.001	0.127	150 (18.0%)	159 (19.1%)	0.571	0.028
Resection or transplantation of long tendon of biceps (23440)	10 (1.2%)	485 (1.4%)	0.632	0.017	10 (1.2%)	10 (1.2%)	1	<0.001
Capsulorrhaphy, glenohumeral joint, posterior, w/ or w/o bone block (23465)	10 (1.2%)	21 (0.1%)	<0.001	0.144	10 (1.2%)	10 (1.2%)	1	<0.001
Capsulorrhaphy, glenohumeral joint, any type multidirectional instability (23466)	10 (1.2%)	19 (0.1%)	<0.001	0.145	10 (1.2%)	10 (1.2%)	1	<0.001
Open treatment of proximal humeral fracture, w/ internal fixation, includes repair of tuberosity(s) (1014066)	50 (6.0%)	1790 (5.1%)	0.277	0.037	50 (6.0%)	51 (6.1%)	0.918	0.005
Open treatment of sternoclavicular dislocation, acute or chronic (1014576)	0 (0%)	15 (0.0%)	0.548	0.029	0 (0%)	0 (0%)	--	--
Open treatment of acromioclavicular dislocation, acute or chronic (1014577)	10 (1.2%)	219 (0.6%)	0.042	0.06	10 (1.2%)	10 (1.2%)	1	<0.001
Open treatment of clavicular fracture, includes internal fixation (23515)	10 (1.2%)	669 (1.9%)	0.13	0.059	10 (1.2%)	10 (1.2%)	1	<0.001
Open treatment of scapular fracture (body, glenoid, or acromion) including internal fixation (23585)	10 (1.2%)	192 (0.6%)	0.014	0.069	10 (1.2%)	10 (1.2%)	1	<0.001
Open treatment of greater humeral tuberosity fracture, includes internal fixation (23630)	10 (1.2%)	256 (0.7%)	0.126	0.047	10 (1.2%)	12 (1.4%)	0.668	0.021
Open treatment of acute shoulder dislocation (23660)	0 (0%)	25 (0.1%)	0.438	0.038	0 (0%)	0 (0%)	--	--
Open treatment of shoulder dislocation, with fracture of greater humeral tuberosity, includes internal fixation (23670)	0 (0%)	33 (0.1%)	0.373	0.044	0 (0%)	0 (0%)	--	--
Open treatment of shoulder dislocation, with surgical or anatomical neck fracture, includes internal fixation (23680)	0 (0%)	33 (0.1%)	0.373	0.044	0 (0%)	10 (1.2%)	0.002	0.156
Medication								
Metformin (6809)	709 (84.9%)	13,833 (39.8%)	<0.001	1.053	708 (84.9%)	727 (87.2%)	0.18	0.066
Insulin (HS501)	514 (61.6%)	11,068 (31.8%)	<0.001	0.625	513 (61.5%)	491 (58.9%)	0.271	0.054

Primary outcomes included shoulder reoperation at one and two years postoperatively. Secondary outcomes were 30-day ED visits and postoperative opioid use, categorized as early (1-30 days), prolonged (30-90 days), and chronic (90-180 days). Opioid outcomes reflected documented prescription activity within each postoperative interval and did not confirm medication consumption. Preoperative opioid exposure could not be standardized and was not directly evaluated within this analysis.

Patient queries, demographic factors, and outcome of interest were identified using current procedural terminology (CPT; a standardized procedural coding system maintained by the American Medical Association), International Classification of Diseases, 10th Revision, Clinical Modification (ICD-10-CM; a standardized diagnostic coding system maintained by the Centers for Disease Control and Prevention), and RxNorm (a standardized medication nomenclature developed by the National Library of Medicine) codes [17-19]. All codes used are provided in Table 3 in the Appendix.

Risk ratios (RR) and 95% confidence intervals (CIs) were computed, and the complication rates were analyzed using the TriNetX system [16]. Categorical variables were assessed using the chi-squared test, while continuous variables were evaluated with independent t-tests. The level of statistical significance was set at p < 0.05.

## Results

Reoperation rates were similar between DPP-4 and non-DPP-4 cohorts at both one year (2.4% vs. 2.5%; RR: 0.952; 95% CI: 0.520-1.744; p = 1.000) and two years postoperatively (3.2% vs. 4.4%; RR: 0.730; 95% CI: 0.449-1.187; p = 0.251). Similarly, 30-day ED visit rates did not differ significantly between groups (2.9% vs. 3.8%; RR: 0.750; 95% CI: 0.446-1.262; p = 0.341).

**Figure 1:**
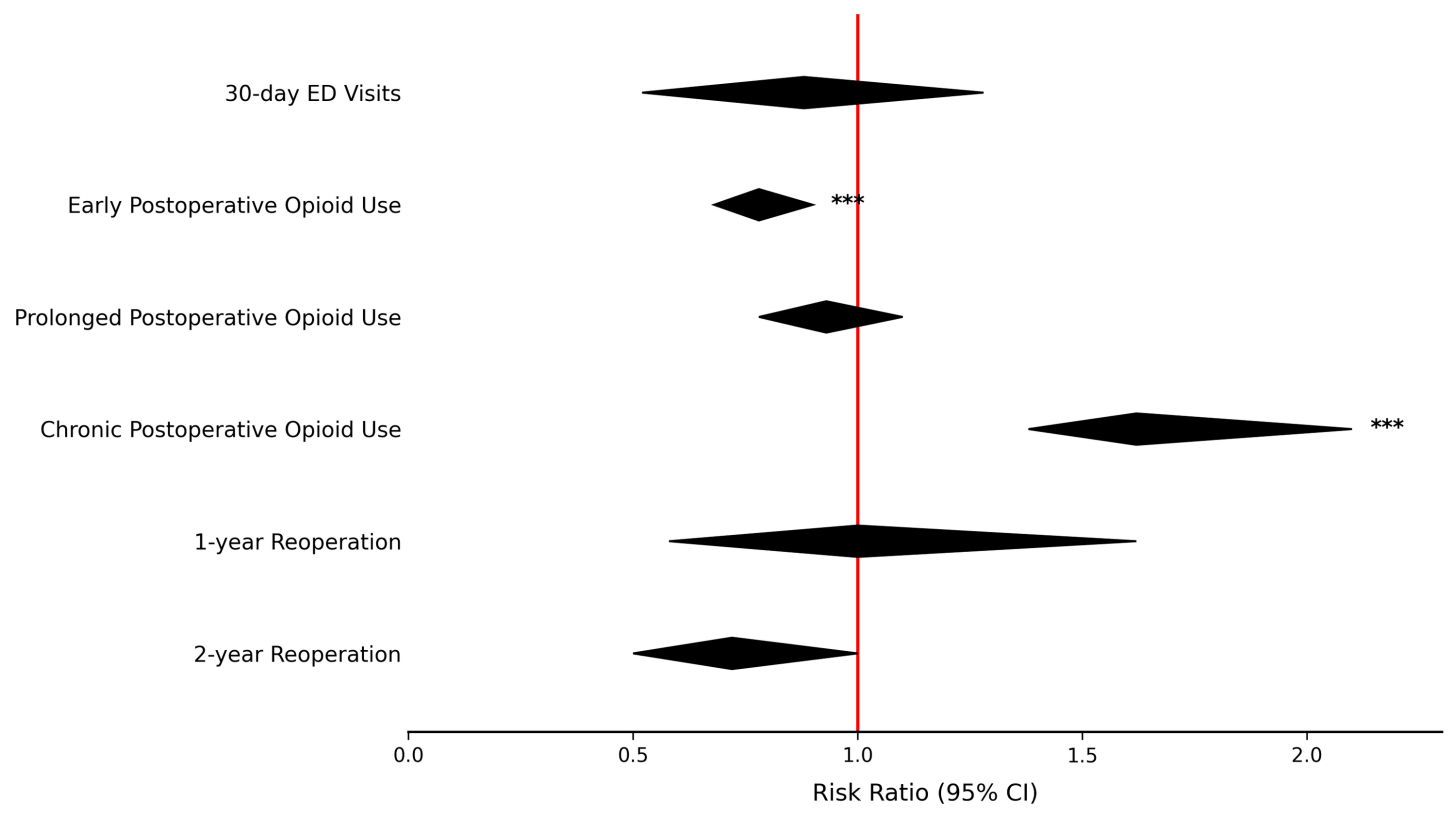
Postoperative outcomes between DPP-4 and non-DPP-4 cohorts following shoulder surgery *** p < 0.001 DPP-4 - dipeptidyl peptidase-4; ED - emergency department

Analysis of postoperative opioid use revealed significant and directionally opposing differences across recovery phases (Table 2, Figure 1). In the early postoperative period (1-30 days), DPP-4 inhibitor users demonstrated substantially lower rates of opioid use compared with non-users (33.6% vs. 45.3%; RR: 0.741; 95% CI: 0.656-0.836; p < 0.001), representing an approximately 26% relative risk reduction. During the prolonged period (30-90 days), opioid use rates were nearly identical between groups (21.5% vs. 21.2%; RR: 1.011; 95% CI: 0.841-1.216; p = 0.952).

**Table 2:** Postoperative outcomes between DPP-4 and non-DPP-4 cohorts following shoulder surgery ED - emergency department; CI - confidence interval

Outcome	DPP-4 events (%)	Non-DPP-4 events (%)	Total	Risk ratio	95% CI	p-value
30-day ED visits	24 (2.9)	32 (3.8)	834	0.750	0.446 – 1.262	0.341
Early postoperative opioid use	280 (33.6)	378 (45.3)	834	0.741	0.656 – 0.836	<0.001
Prolonged postoperative opioid use	179 (21.5)	177 (21.2)	834	1.011	0.841 – 1.216	0.952
Chronic postoperative opioid use	177 (21.2)	114 (13.7)	834	1.553	1.252 – 1.925	<0.001
1-year reoperation	20 (2.4)	21 (2.5)	834	0.952	0.520 – 1.744	1.000
2-year reoperation	27 (3.2)	37 (4.4)	834	0.730	0.449 – 1.187	0.251

In contrast, chronic postoperative opioid use (90-180 days) was significantly higher in the DPP-4 cohort (21.2% vs. 13.7%; RR: 1.553; 95% CI: 1.252-1.925; p < 0.001), representing an approximately 55% relative increase in risk. Notably, the absolute rate of chronic opioid use in DPP-4 users (21.2%) closely approximated that of early opioid use in non-users (21.2%), underscoring the divergence in long-term pain trajectories between groups.

## Discussion

This study investigated the relationship between DPP-4 inhibitor use and postoperative outcomes in patients with type 2 diabetes mellitus undergoing orthopedic shoulder procedures. The main findings were threefold. First, reoperation rates at both one and two years were similar between DPP-4 users and non-users, suggesting that prior DPP-4 exposure does not adversely influence long-term surgical recovery or structural outcomes. Second, short-term emergency medical utilization, measured by 30-day emergency department visits, was comparable between cohorts. Third, patients with DPP-4 inhibitor exposure demonstrated significantly lower early postoperative opioid use but higher chronic opioid use, suggesting a time-dependent relationship between incretin pathway modulation and postoperative pain adaptation. 

The comparable reoperation rates between groups indicate that recent DPP-4 inhibitor exposure does not appear to affect postoperative healing or infection risk to the extent of necessitating additional surgical intervention. This finding aligns with previous literature demonstrating the general surgical safety of incretin-pathway agents in patients with T2DM [20]. Because patients were included based on DPP-4 exposure within six months before surgery, rather than active use at the time of surgery, these findings suggest that prior exposure does not adversely impact long-term surgical outcomes. 

Similarly, short-term emergency medical utilization, as reflected by 30-day ED visits, was comparable between groups. Given that most postoperative ED presentations are related to infection, uncontrolled pain, or metabolic instability, this finding suggests that preoperative exposure to DPP-4 inhibitors does not increase short-term postoperative complications or healthcare utilization. Together, these results demonstrate that patients with recent DPP-4 inhibitor exposure experience postoperative outcomes similar to those of diabetic patients not prescribed these agents. 

The observed reduction in early postoperative opioid requirements among DPP-4 users may reflect the anti-inflammatory and central pain-modulating properties of incretin-based therapies. DPP-4 inhibitors have been shown to attenuate systemic and neuroinflammatory signaling in patients with T2DM [21], which could reduce nociceptive sensitization and thereby decrease acute postoperative pain. These findings align with prior work suggesting that incretin modulation influences inflammatory cascades and immune-neural interactions that shape early pain perception.

Conversely, the increased chronic postoperative opioid use in this cohort is likely multifactorial. Patients prescribed DPP-4 inhibitors are typically older and more medically complex [22], with higher rates of renal impairment and other comorbidities that may predispose them to prolonged opioid utilization. Additionally, early inhibition of inflammatory pathways, while beneficial for acute pain control, may inadvertently interfere with normal healing cascades [23,24], potentially contributing to postoperative stiffness, limited range of motion, or persistent pain states. Although this mechanism remains incompletely characterized in humans, preclinical studies demonstrate that DPP-4 expression is dynamically regulated in glial cells between acute and chronic pain models [25], suggesting a potential link between incretin modulation and long-term nociceptive adaptation. 

Overall, these findings highlight a complex and temporally dependent association between incretin pathway modulation and postoperative pain. While DPP-4 inhibitor exposure was associated with reduced opioid requirements in the immediate postoperative period, it coincided with increased opioid use several months after surgery. This pattern may reflect evolving neuroinflammatory mechanisms, comorbidity-driven pain persistence, or differential recovery trajectories in patients treated with DPP-4 inhibitors. Further research incorporating direct pain and function assessments, as well as laboratory and glycemic data, is warranted to clarify these relationships. 

This study has several limitations. First, as with all retrospective database analyses, there is potential for misclassification or coding inaccuracies. Second, although several comorbidities were controlled for, factors such as concurrent diabetes medications, glycemic control (e.g., HbA1c), and other unmeasured variables may have influenced outcomes. Third, variation in postoperative rehabilitation protocols and medication adherence could not be captured despite matching procedures. Fourth, the lack of patient-reported outcome measures limits our ability to contextualize opioid use with functional recovery or pain etiology during the perioperative period. Fifth, we are unable to confirm the exact durations of DPP-4 inhibitor use and adherence to medication. Sixth, we are unable to calculate exact morphine milligram equivalents for pain medication, which may provide more detailed insight into usage. Finally, preoperative opioid exposure, chronic pain diagnoses, psychiatric comorbidity, diabetic neuropathy severity, and glycated hemoglobin values could not be comprehensively evaluated and may have influenced postoperative opioid utilization. Despite these limitations, the use of a large multicenter propensity score-matched cohort and consistent outcome definitions strengthens the internal validity of this analysis. Although causality cannot be established, the observed findings provide hypothesis-generating evidence regarding postoperative opioid utilization patterns among patients with type 2 diabetes undergoing shoulder surgery.

## Conclusions

DPP-4 inhibitor exposure within six months prior to shoulder surgery was not associated with differences in reoperation rates or early emergency medical utilization among patients with type 2 diabetes. DPP-4 users demonstrated lower early but higher chronic postoperative opioid utilization. These findings should be interpreted as observational associations and support further investigation into factors influencing postoperative pain management in this population.

## Appendices

**Table 3:** CPT, ICD-10-CM, and RxNorm codes for TrinetX queries, inclusions, exclusions, propensity score matching, and outcomes

Diagnosis	Codes
Orthopedic shoulder surgery	1004202, 1004206, 1004209, 1004216, 1004219, 1004224, 1004229, 1014575, 23400, 23415, 23420, 23420, 23440, 23465, 23466, 1014066, 1014576, 1014577, 23515, 23585, 23630, 23660, 23670, 23680, 1005614
Type 2 diabetes mellitus	E11
DPP-4 inhibitors	593411, 857974, 1100699, 1368001
Polytrauma	T07
Exclusion criteria (type 1 diabetes mellitus, systemic connective tissue disorders, long-term corticosteroid use, autoinflammatory syndromes, inflammatory polyarthropathies, prior stress or pathological fractures involving the shoulder or humerus, osteoporosis with current pathological fracture, or prior surgical procedures on the shoulder)	E10, M30-M36, Z79.5, Q79.6, Q87.4, M04, M05-M14, M84.31, M84.32, M84.41, M84.42, M84.51, M84.52, M84.61, M84.62, M80, 1004147, 1005614, 29805
Propensity score inputs	Age at index, female, mle, M81, Z68.3, Z68.4, Z72.0, I50, I10-I1A, E78, I25, 1004202, 1004206, 1004209, 1004216, 1004219, 1004224, 1004229, 1014575, 23400, 23415, 23420, 23430, 23440, 23465, 23466, 1014066, 1014576, 1014577, 23515, 23585, 23630, 23660, 23670, 23680, 1005614, 6809, HS501, E11.2, E11.4
Reoperation	1004147, 1005614, 29805
Opioid Use	5489, 7052, 3423, 7804, 10689
Emergency Department Services	1013711
